# Setting targets for population health improvements: Trends in perinatal health in Europe over the past five years

**DOI:** 10.1093/eurpub/ckac129.110

**Published:** 2022-10-25

**Authors:** K Szamotulska, M Loghi, G Weber, G Heller, I Zile-Velika, J Isakova, K Monteath, M Jané Checa, WH Zhang, M Gissler

**Affiliations:** 1 Department of Epidemiology, Institute of Mother and Child, Warsaw, Poland; 2 Directorate for Social Statistics & Welfare, Italian Statistical Institute, Rome, Italy; 3 Department of Epidemiology and Statistics, Directorate of Health, Ministry of Health, Luxembourg, Luxembourg; 4 Institute for Quality Assurance and Transparency in the Healthcare Sector, Berlin, Germany; 5 Center for Disease Prevention and Control, Riga, Latvia; 6 Health Statistics Department, Institute of Hygiene, Vilnius, Lithuania; 7 Public Health Scotland, Edinburgh, UK; 8 Department of Health, Public Health Agency of Catalonia, Barcelona, Spain; 9 School of Public Health, Université Libre de Bruxelles, Brussels, Belgium; 10 Finnish Institute for Health and Welfare, Helsinki, Finland

## Abstract

**Background:**

The Euro-Peristat network documented disparities in perinatal outcomes between countries in Europe in its reports published every 5 years, but trend analyses were limited because data were not collected annually.

**Methods:**

Using the Euro-Peristat PHIRI protocol, we estimated rates and assessed trends between 2015 and 2019 for preterm birth, stillbirth, neonatal mortality and caesarean delivery. Country-specific relative risks (RR) for year, modelled as a continuous variable, were estimated and random effects meta-analysis used to generate pooled RRs. Heterogeneity was measured with the I2 statistic (percentage of variability in estimates due to heterogeneity rather than sampling error).

**Results:**

Stillbirth rates ≥24 weeks of gestational age (GA) varied in 2019 from <2.5 per 1000 births in Denmark, Estonia, Finland and Slovenia to over 4 per 1000 in Belgium, Cyprus, UK Wales and Lithuania. Preterm birth rates ranged from <6% in Lithuania, Finland, Latvia, Estonia and Denmark to 8% or more in Portugal, Belgium, UK Scotland and Cyprus. Fewer than 20% of births were by caesarean in Norway, the Netherlands, Finland, Estonia in comparison to one-third in Cyprus, Ireland, Italy, UK Scotland. Trends over time differed between countries and were not related to the level of the indicator: the pooled RR by year for preterm birth was 0.99 [0.99; 1.00] with five countries having significant decreases and three countries having increases. Caesarean section rates were stable overall (RR: 1.00 [0.99; 1.01]RR:1.00, 95% CI: 0.99-1.01), but with high heterogeneity (I2=99%); in six countries rates increased significantly, whereas in nine rates decreased between 2015 and 2019.

**Conclusions:**

European countries have varying rates and trends of the principal perinatal health indicators. Investigation of policies in high-performing countries could provide guidance for improvement elsewhere.

